# Anti-Rickettsial Activity of Chitosan, Selenium, and Silver Nanoparticles: Efficacy in Vero Cells

**DOI:** 10.3390/pathogens14090885

**Published:** 2025-09-04

**Authors:** Yevheniy-Yuliy Peresh, Zdenko Špitalský, Mohamed Shaalan, Eva Špitalská

**Affiliations:** 1 Institute of Virology, Biomedical Research Center, Slovak Academy of Sciences, Dúbravská cesta 9, 845 05 Bratislava, Slovakia; virupere@savba.sk; 2 Institute of Polymer, Slovak Academy of Sciences, Dúbravská cesta 9, 845 41 Bratislava, Slovakia; zdeno.spitalsky@savba.sk (Z.Š.); shaalan@savba.sk (M.S.); 3 Department of Pathology, Faculty of Veterinary Medicine, Cairo University, Giza 12211, Egypt

**Keywords:** *Rickettsia* species, tick-borne pathogen, tick, host cells, nanoparticles, therapy

## Abstract

Nanoparticles have emerged as innovative tools for combating bacterial infections, offering a potential solution to antibiotic resistance and the limitations of conventional antimicrobials. Nanoparticles exhibit antibacterial activity through multiple mechanisms, including oxidative stress induction, metal ion release, direct membrane damage, disruption of DNA and proteins, and indirect immune system enhancement. *Rickettsia helvetica*, *R. monacensis*, *R. slovaca*, and *R. conorii* subsp. *raoultii* are tick-borne pathogens transmitted by *Ixodes ricinus*, *Dermacentor reticulatus*, and *D. marginatus* ticks across Europe causing spotted fever rickettsiosis. While rickettsioses are successfully treated with antibiotics, resistance of rickettsiae to antimicrobial therapy has been reported. Here, we evaluated the anti-rickettsial activity of silver (AgNPs), selenium (SeNPs), and chitosan (CSNPs) nanoparticles against *R. conorii* subsp. *caspia*, a tick-borne bacterial pathogen, in African green monkey kidney cell line (Vero). At their highest non-cytotoxic concentrations, CSNPs exhibited the strongest inhibitory effect (87%). SeNPs also significantly reduced bacterial load (76%), although their efficacy was constrained by cytotoxicity at higher doses. In contrast, AgNPs did not show significant activity under the tested conditions. The differences observed among nanoparticles reflect both the antimicrobial properties and host cell tolerance limits. These findings highlight CSNPs and SeNPs as promising candidates for further development of nanoparticle-based strategies to combat intracellular, tick-borne pathogens.

## 1. Introduction

Rickettsiae, measuring 0.3 to 0.5 × 0.8 to 2.0 μm, are obligate intracellular, pleomorphic, aerobic, gram-negative bacteria with a life cycle confined to host cells, predominantly infecting and replicating within endothelial cells. They are typically localized in the cytoplasm; however, species from the spotted fever group (SFG) have also been observed within the host cell nucleus [1,2]. The rickettsial cell envelope consists of an outer membrane, an inner cytoplasmic membrane, and a microcapsular layer. The outer membrane contains proteins, phospholipids, and lipopolysaccharides (LPSs). These LPSs are heat-stable, immunogenic, and group-specific antigens that play a key role in activating nonspecific antibacterial defenses. Their presence contributes to the high endo-toxic potential of rickettsiae [3,4,5]. Originally, bacteria of the genus *Rickettsia* were classified based on morphological, antigenic, and metabolic characteristics into two major groups: SFG, which includes species transmitted primarily by hard ticks (e.g., *Rickettsia conorii*—the causative agent of Mediterranean spotted fever (MSF), *R. rickettsia*—the agent of Rocky Mountain spotted fever, *R. helvetica* and *R. monacensis*, *R. slovaca*, *R. conorii* subsp. *raoultii,* and others) and the typhus group (TG) responsible for epidemic and endemic typhus [6]. *Rickettsia helvetica* and *R. monacensis* are tick-borne pathogens transmitted by *Ixodes ricinus* and are associated with MSF-like rickettsioses. *Rickettsia slovaca* and *R. conorii* subsp. *raoultii,* transmitted by *Dermacentor reticulatus* and *D. marginatus* ticks, are the causative agents of tick-borne lymphadenopathy (TIBOLA). These species are distributed throughout Europe, including Slovakia. MSF, caused by *R. conorii* subsp. *conorii*, was first described in 1932 and is clinically characterized by fever, an inoculation eschar, and a maculopapular rash. In severe cases, complications such as myocarditis, retinopathy, meningitis, pancreatitis, or renal failure may occur. The disease has an incubation period of approximately six days and a reported mortality rate of up to 13% [7,8]. *Rickettsia conorii* subsp. *caspia* is the etiological agent of Astrakhan fever (AF), so named after the region where the first cases were identified, near Astrakhan and the Caspian Sea. Clinical manifestations closely resemble those of MSF, including high fever, eschar (in ~23% of cases), a maculopapular rash (91%), and petechiae (20%) [8]. The primary vector of *R. conorii* subsp. *caspia* is the tick *Rhipicephalus pumilio*. In Europe, this rickettsial subspecies has also been detected in *Rhipicephalus sanguineus* ticks in regions such as Kosovo and southern France, suggesting AF may represent an underdiagnosed cause of spotted fever syndromes in Europe and that its geographic distribution may extend beyond the Astrakhan region [7,8]. Doxycycline remains the first-line treatment for infections caused by rickettsiae, while certain antibiotics are contraindicated in these cases. Nanoparticles with intrinsic antimicrobial properties represent a promising alternative to conventional antibiotics or may serve as prophylactic agents in the early stages of tick-borne infections.

Nanoparticles are emerging as valuable tools for both the diagnosis and treatment of rickettsial infections, particularly through rapid assays and targeted drug delivery systems. Willson et al. (2025) [9] used europium chelate nanoparticles in a lateral flow assay to detect a biomarker (the putative N-acetylmuramoyl-L-alanine amidase RC0497) specific to *Rickettsia rickettsii* and *R. conorii*. The assay demonstrated 95.5% sensitivity and 100% specificity in infected animal models, offering a promising tool for the rapid diagnosis of SFG rickettsioses. Velásquez et al. (2024) [10] developed a poly(lactic-co-glycolic acid) (PLGA) nanoparticle system functionalized with salmon IgM to deliver the antibiotic florfenicol against *Piscirickettsia salmonis*. The system reduced bacterial load in macrophages, indicating potential for targeted drug delivery in aquaculture. Acedo-Valdez et al. (2017) [11] proposed nanoparticle-based antibacterial strategies to reduce mortality and bacterial nodules in shrimp infected with necrotizing hepatopancreatitis bacterium, a rickettsia-like organism, using biosynthesized silver nanoparticles. Peresh et al. (2024) [12] demonstrated the antibacterial activity of hydrophilic and hydrophobic carbon quantum dots against *Rickettsia slovaca*, suggesting that citric acid-based carbon quantum dots were a promising candidate as anti-rickettsial agents. Despite a few recent studies, the antibacterial and antiviral potentials of nanoparticles in the context of tick-borne infections remain a largely unexplored area of research. Therefore, this study aimed to analyze and compare the antimicrobial effects of synthesized silver (AgNPs), selenium (SeNPs), and chitosan (CSNPs) nanoparticles against *R. conorii* subsp. *caspia* infections in vitro using an animal host cell line (Vero).

## 2. Materials and Methods

### 2.1. Cultivation of R. conorii subsp. caspia

*Rickettsia conorii* subsp. *caspia* was obtained from the collection of the Department of Rickettsiology at the Institute of Virology, Biomedical Research Center of the Slovak Academy of Sciences. The strain was revived using a Vero cell line (*Cercopithecus aethiops* monkey epithelial cells ATCC^®^ CCL-81™, Manassas, VA, USA) cultured in Dulbecco’s modified Eagle’s medium (DMEM) (Biosera, Cholet, France) supplemented with 3% fetal bovine serum (FBS) at 34 °C in a humidified atmosphere containing 5% CO_2_. Confluent monolayers of cell lines in a 12-well plate with a lid were infected with rickettsial particles and incubated for 24 hours (h).

### 2.2. Nanoparticles Synthesis

The synthesis of AgNPs was conducted by the chemical reduction method [13]. Silver nitrate (AgNO_3_) (Sigma Aldrich, Darmstadt, Germany) was subjected to reduction reaction with the aid of trisodium citrate and sodium borohydride (Sigma Aldrich, Darmstad, Germany) as reducing agents. Polyvinylpyrrolidone (PVP) (Sigma Aldrich, Darmstad, Germany) was added to enhance the stability of silver nanoparticles and prevent their aggregation. The synthesized AgNPs were stored in the refrigerator at 4 °C and covered with aluminum foil to protect from exposure to light [14]. SeNP synthesis was carried on preparing an aqueous solution of sodium selenite (100 mM) (Sigma Aldrich, Darmstad, Germany). Ascorbic acid (50 mM) (Sigma Aldrich, Darmstad, Germany) was added at the ratio of 1:2 in a dropwise manner to the sodium selenite aqueous solution with continuous stirring for 30 min [15]. CSNPs were synthesized using the ionic gelation method. Briefly, low molecular weight chitosan, deacetylation ≥ 75% (0.5% *w*/*v*) (Sigma Aldrich, Darmstad, Germany), was dissolved in dilute aqueous acetic acid (1%) (CentralChem, Bratislava, Slovakia). Then, aqueous solution of sodium tripolyphosphate salt (0.25%, Sigma Aldrich, Hamburg, Germany) was added drop by drop at the ratio of 1:3 (*v*/*v*). The pH was adjusted by NaOH (10 N) to 4.7 ± 0.1. The obtained suspension was centrifuged at 4000× *g* for 30 min at 4 °C and stored in a refrigerator [16]. The characterization of AgNPs, SeNPs, and CSNPs were separately detailed in our previous publications [14,15,16].

### 2.3. Cell Viability

The 3-(4,5-dimethylthiazol-2-yl)-2,5-diphenyltetrazolium bromide (MTT) cell viability assay was used to determine the non-cytotoxic concentration of the nanoparticles (NPs) in both cell lines. Briefly, 10^4^ cells per well were seeded in a 96-well plate and incubated in DMEM supplemented with 10% FBS at 37 °C in a 5% CO_2_ atmosphere for 24 h. Afterward, the medium was replaced with DMEM containing 3% FBS and the respective NPs. The cells were then cultured for an additional 24 h. For the MTT assay of mitochondrial dehydrogenase activity, cells were incubated at 37 °C for 3 h with 0.5 mg/mL of tetrazolium salt MTT. Following incubation, the medium was removed, and the resulting formazan crystals were dissolved in DMSO. The color development was measured at 570 nm using a microplate reader.

In the CellTiter-Blue cell viability assay, a buffered solution of resazurin was added to the wells according to the manufacturer‘s instructions. After a 3 h incubation, fluorescence was recorded at 560/590 nm.

In both assays, NPs were tested in hexaplicates. Only DMEM with 3% FBS was added to the control wells. The concentrations used in the tests were as follows: serial dilutions of 1.7 × 10^−1^ to 1.7 × 10^−8^ mg/mL for SeNPs, 1.73 × 10^−3^ to 1.73 × 10^−7^ mg/mL for AgNPs, and 5 × 10^−1^ to 5 × 10^−8^ mg/mL for CSNPs.

For the study of the effectiveness of NPs on rickettsial infections concentrations with cytotoxicity not exceeding 10%, as determined by cell viability assays were used.

### 2.4. Treatment of Rickettsial Infection with Nanoparticles

Twenty-four hours post-inoculation (p.i.), NP treatment was initiated. NPs were applied to confluent monolayers of host cells for a duration of 24 h and subsequently administered at 24 h intervals. Control experiments were conducted in parallel without NP treatment. All experiments were performed in triplicate for both host cell lines. The effectiveness of treatment was evaluated after 24, 48, and 72 h.

### 2.5. RNA Extraction and Reverse-Transcriptase Quantitative PCR

To quantify viable *R. conorii*, quantitative real-time PCR (qPCR) was conducted. Total RNA and DNA were extracted using the AllPrep DNA/RNA Mini Kit (Qiagen, Hilden, Germany). RNA samples were treated with RNase-Free DNase (Qiagen, Hilden, Germany) and further purified using the RNeasy MinElute Cleanup Kit (Qiagen, Hilden, Germany). Complementary DNA (cDNA) was synthesized from 100 μg of purified RNA using random hexamer primers and the First Strand cDNA Synthesis Kit (Thermo Scientific, Waltham, MA, USA), according to the manufacturer’s instructions. No-reverse transcriptase controls and negative controls (nuclease-free water) were included to confirm the specificity of cDNA synthesis. The concentration and purity of RNA and DNA were measured with a Nanophotometer (Implen, Westlake Village, CA, USA). Samples were stored at −20 °C until further analysis. Rickettsial copy numbers were quantified using TaqMan-based qPCR targeting a 74- bp fragment of the citrate synthase-encoding gene (*gltA*) [17]. Amplification was carried using SuperHot Master Mix (2×) (Bioron, Römerberg, Germany) on a Bio-Rad CFX96™ Real-Time PCR System (Bio-Rad, Hercules, CA, USA). Thermal cycling conditions consisted of an initial denaturation at 95 °C for 3 min, followed by 40 cycles of denaturation at 94 °C for 20 s and combined annealing/extension at 60 °C for 40 s, with fluorescence data collected in single acquisition mode. Each qPCR run included a no-template control and standards. A standard curve was generated from serial dilutions (3 × 10^1^ to 3 × 10^6^ copies) of *R. slovaca* DNA. All samples were analyzed in technical duplicates.

### 2.6. Statistical Analysis

The Mann–Whitney U test was used to compare the number of *Rickettsia gltA* gene copies in viable bacterial cells between treated wells and control wells [18]. Results with *p* values of < 0.05 were considered statistically significant. The effectiveness of NPs was calculated as the percentage reduction in bacterial load using the following formula: R (%) = [(a − b)/a] × 100, where a represents the rickettsial copy number in the control sample, and b represents the rickettsial copy number in the treated sample [19].

## 3. Results

To assess the effect of NPs on rickettsial inactivation, the highest non-toxic concentrations for host cells, as determined by cell viability assays and not exceeding 10%, were used: 1.7 × 10^−4^ mg/mL for SeNPs, 1.73 × 10^−4^ mg/mL for AgNPs, and 5 × 10^−1^ mg/mL for CSNPs.

**Figure 1:**
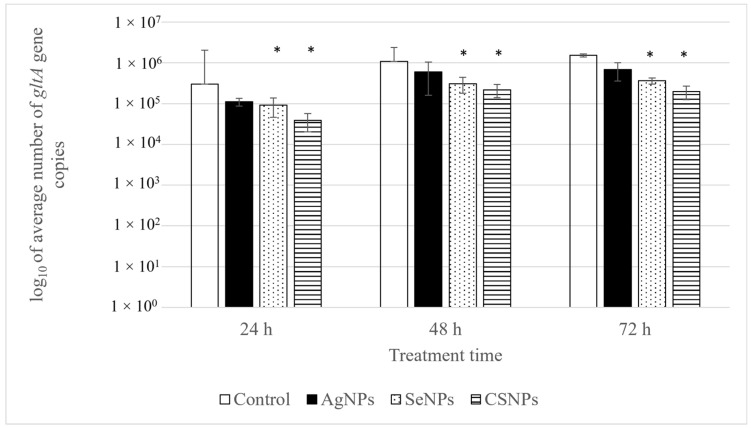
Numbers of *Rickettsia conorii gltA* gene copies after 24, 48, and 72 h treatments with nanoparticles in infected Vero cells. *p* < 0.05 is referred to as statistically significant and is marked with *.

The impact of NPs on the inactivation of *R. conorii* infection was evaluated in a mammalian epithelial cell line Vero using qPCR at 24, 48, and 72 h intervals. The corresponding growth curves of *R. conorii* in treated and untreated cells are presented in Figure 1. The effectiveness of NPs, expressed as the percentage reduction in bacterial load (R) is presented in Table 1.

CSNPs exhibited the most pronounced inhibitory effect on *R. conorii* infection in Vero cells. At 24, 48, and 72 h post-treatment, the reduction reached 87.14%, 79.67%, and 87.09%, respectively. All effects were statistically significant (*p* = 0.00512), confirming the strong inhibitory activity of CSNPs.

SeNPs exhibited a statistically significant anti-rickettsial effect at all time points examined, although the magnitude of inhibition was consistently lower than that observed for CSNPs. Reductions of 69.61%, 71.27%, and 76.20% were observed at 24, 48, and 72 h post-treatment, respectively (*p* < 0.05 at all time points). These results demonstrate that SeNPs exert a progressive, time-dependent inhibitory effect on rickettsial proliferation, although their overall efficacy is lower compared with CSNPs.

AgNPs showed the lowest efficacy against *R. conorii* infection in Vero cells. Although reductions in rickettsial copy numbers were observed, these changes were not statistically significant. Reductions of 63.11%, 44.50%, and 55.31% were recorded at 24, 48, and 72 h, respectively. These data indicate that AgNPs were unable to achieve consistent or statistically significant suppression of intracellular rickettsial proliferation under the tested conditions.

## 4. Discussion

Nanoparticles have demonstrated promising antibacterial effects, including activity against intracellular bacteria, which are difficult to treat due to their ability to evade the host-immune response by residing within the host cell. In our study we used three different nanoparticles: AgNPs, SeNPs, and CSNPs. These NPs have shown broad-spectrum antibacterial activity, including against multidrug-resistant strains such as *Staphylococcus aureus*, *Salmonella typhimurium*, *Pseudomonas aeruginosa*, *Stenotrophomonas pavanii*, and *Aeromonas enteropelogenes* [20,21,22,23,24,25]. Their mechanism of action primarily involves the induction of oxidative stress through reactive oxygen species (ROS), which damage essential biomolecules such as proteins, DNA, and lipids [24,26,27,28,29]; the disruption of membrane integrity; and the depletion of intracellular ATP—while maintaining low cytotoxicity to host cells, making them suitable for intracellular applications [30,31,32,33,34,35]. Moreover, SeNPs act synergistically with antimicrobial agents, such as lysozyme or antibiotics, to enhance bacterial inhibition and reduce resistance development [33,36,37,38]. Similarly, hybrid formulations—such as CSNPs combined with cationic peptides or antibiotics—have demonstrated enhanced specificity and potency against intracellular bacteria while maintaining low host toxicity [39,40]. Examples include ceftriaxone-, gentamicin-, and levofloxacin-loaded CSNPs, as well as nitrogen-phosphorous-carbonized chitosan nanoparticles, all of which significantly reduce intracellular *Salmonella typhimurium*, *Brucella*, and *Staphylococcus aureus* in macrophages and epithelial cells [39,40,41,42].

In this study, NPs were applied to combat *R. conorii* subsp. *caspia* infection of mammalian epithelial Vero cells. Both CSNPs and SeNPs demonstrated statistically significant anti-rickettsial activity, although their efficacy differed. The highest antibacterial efficacy was achieved with CSNPs, reaching 87% inhibition, whereas SeNPs reduced rickettsial infection by 76%. Their activity reflects both nanoparticle potency and the concentrations that could be safely applied to host cells. CSNPs exhibited a stronger inhibitory effect overall, whereas SeNPs were less potent but tested at lower, non-cytotoxic concentrations. Notably, increasing SeNPs concentrations was not feasible due to host cell toxicity, while CSNPs were used at relatively high non-toxic doses. These observations suggest that the apparent differences in efficacy are influenced not only by the activity of the nanoparticles but also by constraints imposed by the host cells. It is probable that CSNPs at lower concentrations, comparable to those used for SeNPs, would exhibit reduced anti-rickettsial activity, further emphasizing the importance of optimizing nanoparticle type and dosing.

AgNPs, unlike CSNPs and SeNPs, demonstrated only a modest and statistically non-significant effect on the intracellular growth of *R. conorii* in Vero cells. Although partial reductions in bacterial load were observed at different time points, the variability and lack of significance suggest that AgNPs alone are unlikely to provide sufficient therapeutic benefits against obligate intracellular pathogens. Nonetheless, their known ability to synergize with conventional antibiotics and to enhance drug delivery into host cells supports the possibility of employing AgNPs in combinatorial treatment strategies, rather than as a primary anti-rickettsial agent. While most studies focus on extracellular bacteria, the ability of AgNPs to penetrate host cells and induce intracellular damage suggests potential applicability against intracellular pathogens. However, selective toxicity and host cell safety remain key challenges [26,28,43]. Furthermore, combining AgNPs with antibiotics such as erythromycin, carbicillin, or moxifloxacin has been shown to enhance antibacterial activity, particularly against resistant strains like *Streptococcus mitis* and *Pseudomonas aeruginosa* [44]. Therefore, while AgNPs alone cannot be considered a reliable anti-rickettsial treatment, their incorporation into well-designed combinatorial or nanocarrier-based strategies could represent a feasible approach to enhance therapeutic efficacy while minimizing host cell toxicity.

The results highlight the potential of biocompatible nanoparticles as alternative or adjunct strategies for controlling rickettsial infections. In this study, only a single pathogen, *R. conorii*, was tested, so the observed anti- *R. conorii* effects could not be generalized and would need to be further validated. However, AgNPs, SeNPs, and CSNPs represent promising tools for combatting infections caused by both extracellular and intracellular bacteria. Their application against tick-borne intracellular pathogens, such as *Rickettsia*, remains underexplored but has shown substantial promise, warranting further investigation into their effect in different host cells, underlying mechanisms, and clinical potential. In addition, by demonstrating effective inhibition of rickettsial proliferation in vitro, this study provides a foundation for future in vivo investigations, which are essential to determine the therapeutic window, pharmacodynamics, and safety profile of these nanomaterials. Overall, the work underscores the importance of balancing nanoparticle potency with host cell tolerance to maximize efficacy while minimizing cytotoxicity, offering valuable insights for the development of nanoparticle-based prophylactic or therapeutic approaches against rickettsial pathogens.

## 5. Conclusions

This study shows that biocompatible nanoparticles have strong potential as anti-rickettsial agents. CSNPs demonstrated the most pronounced inhibitory effect on *R. conorii* proliferation in Vero cells, while SeNPs also showed significant but lower activity, limited by host cell cytotoxicity at higher concentrations. AgNPs did not exhibit significant activity under the tested conditions. These findings emphasize the need to optimize nanoparticle type and dosing, balancing antimicrobial efficacy with host cell tolerance. Overall, this work provides the first evidence that CSNPs and SeNPs can substantially reduce rickettsial burden in vitro, supporting their further development as alternative or adjunct therapies against intracellular, tick-borne pathogens, and highlighting the directions in which future studies should be focused.

## Figures and Tables

**Table 1:** Percentage reduction (R) in bacterial load between control and treated samples.

Treatment Time (h)	Bacterial Load in Untreated Cells (Average Number of *gltA* Gene Copies/μL)	Bacterial Load in Treated Cells (Average Number of *gltA* Gene Copies/μL)	R (%)
CSNPs			
24	3.00 × 10^5^	3.86 × 10^4^	87.14
48	1.08 × 10^6^	2.20 × 10^5^	79.67
72	1.53 × 10^6^	1.98 × 10^5^	87.09
SeNPs			
24	3.00 × 10^5^	9.13 × 10^4^	69.61
48	1.08 × 10^6^	3.10 × 10^5^	71.27
72	1.53 × 10^6^	3.65 × 10^5^	76.20
AgNPs			
24	3.00 × 10^5^	1.11 × 10^5^	63.11
48	1.08 × 10^6^	6.00 × 10^5^	44.50
72	1.53 × 10^6^	6.86 × 10^5^	55.31

## Data Availability

The authors confirm that the data supporting the findings of this study are available within the article.

## Abbreviations

The following abbreviations are used in this manuscript:

AgNPs	Silver nanoparticles
SeNPs	Selenium nanoparticles
CSNPs	Chitosan nanoparticles
ROS	Reactive oxygen species
SFG	Spotted fever group
TG	Typhus group
